# Effects of Aspect on Clonal Reproduction and Biomass Allocation of Layering Modules of *Nitraria tangutorum* in Nebkha Dunes

**DOI:** 10.1371/journal.pone.0079927

**Published:** 2013-10-29

**Authors:** Qinghe Li, Jun Xu, Huiqing Li, Saixiao Wang, Xiu Yan, Zhiming Xin, Zeping Jiang, Linlong Wang, Zhiqing Jia

**Affiliations:** 1 Research Institute of Forestry, Chinese Academy of Forestry; Key Laboratory of Tree Breeding and Cultivation, State Forestry Administration, Beijing, China; 2 Experimental Center of Desert Forestry, Chinese Academy of Forestry, Dengkou, Inner Mongolia Autonomous Region, China; 3 Institute of Desertification Studies, Chinese Academy of Forestry, Beijing, China; Lawrence Berkeley National Laboratory, United States of America

## Abstract

The formation of many nebkha dunes relies on the layering of clonal plants. The microenvironmental conditions of such phytogenic nebkha are heterogeneous depending on the aspect and slope. Exploring the effects of aspect on clonal reproduction and biomass allocation can be useful in understanding the ecological adaptation of species. We hypothesized that on the windward side layering propagation would be promoted, that biomass allocation to leaves and stems of ramets would increase, and that the effects of aspect would be greater in the layering with larger biomass. To test these hypotheses, we surveyed the depth of germination points of axillary buds, the rate of ramet sprouting, the density of adventitious root formation points, and the biomass of modules sprouting from layering located on the NE, SE, SW and NW, aspects of *Nitraria tangutorum* nebkhas. The windward side was located on the NW and SW aspects. The results indicated that conditions of the NW aspect were more conducive to clonal reproduction and had the highest rate of ramet sprouting and the highest density of adventitious formation points. For the modules sprouting from layering on the SW aspect, biomass allocation to leaves and stems was greatest with biomass allocation to adventitious roots being lowest. This result supported our hypothesis. Contrary to our hypothesis, the effects of aspect were greater in layering of smaller biomass. These results support the hypothesis that aspect does affect layering propagation capacity and biomass allocation in this species. Additionally, clonal reproduction and biomass allocation of modules sprouting from layering with smaller biomass was more affected by aspect. These results suggest that the clonal growth of *N. tangutorum* responses to the microenvironmental heterogeneity that results from aspect of the nebkha.

## Introduction

Nebkhas are composed of wind-borne sediment within or around the canopies of plants and are an adaptive feature of the growth of plants in arid and semiarid regions [1], [2], [3]. These phytogenic mounds are the habitat of many clonal plants that perform advantageous clonal reproduction [4], [5] through layering modules. The whole clonal plant can increase its survival and regenerate through layering. In layering, plants sprouts adventitious roots downwards and ramet shoots upwards coinciding with burial events. The biomass allocation of clonal plants to different plant organs can be used as an indicator of resource availability and amount of disturbance. The formation of nebkha creates microenvironments with different microclimate conditions and amounts of sand movement. Although many studies have mentioned the microenvironmental condition of the dune system [1], [6], [7], [8], this component of a nebkha has attracted little attention. The microenvironmental conditions on different aspects of a clonal plant nebkha may have marked effects on the growth and development of the plants.

The nebkhas of woody plants are formed as a function of wind regime, biological factors, and sand supply. Sand movement is very sensitive to the direction of wind, especially the prevailing wind directions [8], [9], [10], [11], which display sand erosion on the windward slope and sand accretion on the leeward slope [8], [10], [12]. The clonal plant nebkhas are exposed to frequent disturbance by aeolian sand activity [11], [13], [14]. The ramets of clonal plants are often exposed to different amounts of wind erosion and sand burial on different aspects of one nebkha [12], [15], [16], [17], [18]. Sand burial in particular is of great importance to asexual reproduction via its impact on seedling emergence, root suckers, and layering [19], [20], [21], [22]. Previous studies have indicated that there were a variety of effects of sand burial on the clonal growth in plant. The relatively shallow and short-term sand burial may promote the growth of some plants in terms of shoot height, total plant biomass, and/or the number of ramets. However, ramet number, leaf number, and biomass per plot decreased with increasing burial level [23], [24], [25], [26], [27]. Plants growing on the windward side commonly lose water from the root system due to wind erosion exposing root tissues [18]. On the leeward side, the shoots of ramets are prone to be buried by sand and surviving ramets grow through the sand deposit by elongating the stem, increasing the number of nodes and the length of internodes. This elongation occurs at the expense of the root system, indicating that available energy was re-allocated to above-ground parts [14], [25], [26], [28]. Increasing sand burial can compact the soil which creates a physical barrier to the emergence of shoots [29]. Additionally, microclimate conditions on the microsites of the nebkhas were substantially different [1]. The aspect-relative heterogeneity in soil microclimate within such phytogenic nebkha may be important in determining the success of the species. Soil moisture is a key limiting factor for the clonal growth in desert plants. Site exposure (aspect) influences the surface moisture dynamics (relative to the evapotranspiration and soil heat flux), thereby affecting energy transfer efficiency between plants and the environment [30]. Soil moisture loss is slower on the north facing slope due to solar angle. The patterns of species composition were correlated with the spatial variability in soil moisture [1], [31]. The cutting germination decreased mainly as a function of the decrease in soil humidity [32]. These heterogeneous soil microclimate conditions may eventually affect clonal reproduction and biomass allocation of clonal plants.

The size of clonal structures is an important factor in the clonal growth of plants. Some studies have mentioned clonal fragments under burial [27], [29], [33] and found that larger fragments have a greater ability to respond to favorable conditions of horizontal position. This is due to the amount of stored resources contained in a fragment [29], [33]. The vegetative regeneration of plants may be directly determined by the clone properties, e.g. buds on the layering that are the primary shoot-producing meristematic organs [34], [35], [36], [37], [38]. The clonal growth of a size-dependent clone is common [33], [38], [39], [40], [41]. One study found that the big *Glechoma hederacea* clone developed a greater number of ramets under favorable conditions [38]. However, little is known about the effect of layering size.

This study builds upon previous studies by asking how the aspect of a clonal plant nebkha influences clonal reproduction and biomass allocation of *Nitraria tangutorum* found in a river deposit in northeastern Ulan Buh Desert, China. We hypothesized (1) that, once buried on the windward direction, layering propagation capacity would increase and biomass allocation to sprouted leaves and stems would increase while allocation to adventitious roots would decrease and (2) that effects of aspect will be greater in the layering with greater biomass. We investigated the clonal reproduction and biomass of the northeast, southeast, southwest, and northwest facing sides of *N. tangutorum* nebkhas to test these hypotheses.

## Materials and Methods

### Ethics Statement

The study site is maintained by the Experimental Center of Desert Forestry of the Chinese Academy of Forestry, which is located in the northeastern Ulan Buh Desert of Northwestern China. It is an experimental base for the researchers of the Chinese Academy of Forestry. Thus we could conduct experiments there without any specific permits. The field study did not involve endangered or protected species.

### Research Species

*Nitraria tangutorum* Bobr, a plant unique to China, is a xerophyte mainly distributed in Inner Mongolia, Xinjiang, Qinghai, Ningxia, and other places of China. It exhibits strong wind sheltering and sand stabilization, drought resistance, heat resistance, salt tolerance, and barren endurance. *N. tangutorum* mainly grows in clay covered with aeolian sand in the arid deserts and desert steppes. It is a dominant species important in structuring desert plant communities [42]. *N. tangutorum* can reproduce both sexually and asexually, but seeds rarely form new plants under natural conditions due to a thick and hard seed coat combined with negative effects of drought, sandstorms, and other severe conditions. When a branch is buried in the sand, layering occurs. Some adventitious roots may be produced and some axillary buds in the layering will develop into ramets when there is sufficient moisture and the burial depth is appropriate. Therefore, clonal reproduction is the primary reproductive strategy of *N. tangutorum*.

*N. tangutorum* creates branch-derived clonal offshoots which are propagated through the process of layering. Nebkhas of various sizes eventually come into being through sand aggregation creating the unique landscapes of the arid desert. A *N. tangutorum* nebkha is the result of years of sand deposition, and the original ortet that germinated from seeds is deeply buried and may even be decayed. As a result, a *N. tangutorum* plant on a nebkha emerges as an integrated whole from layering modules, adventitious root modules, leaf modules, and stem modules.

### Study Area

Ulan Buh Desert, located in the central zone of the northern sandland series of China, is a temperate arid desert. The field experiment was performed in the northeastern region of Ulan Buh Desert and the southwestern part of Houtao Plain in the middle reaches of the Yellow River, which is seated in Dengkou County, Bayan Nur City, Inner Mongolia Autonomous Region. The average annual precipitation is 144.5 mm (1954 to 2003), annual evaporation is about 2380.6 mm, and the average annual temperature is approximately 7.8°C. The region is characterized by a temperate continental monsoon climate with prevailing westerly and northwesterly winds, the latter being the principal damaging wind. This study was conducted in section of a river deposit with a large number of *N. tangutorum* nebkhas. The field site is dominated by a transitional soil (from desert to desert steppe) with incomplete zonal development. The soil consists of clay deposited by the river and scattered sandy soil stabilized by *N. tangutorum* nebkhas. Often there are cracks in the clay as a result of rare precipitation. The associated plant species mainly grow in the inter-dune area and include *Artemisia ordosica*, *A. sphaerocephala*, *Agriophyllum squarrosum*, *Salsola beticolor*, *Corispermum mongolicum*, and *Psammochloa villosa*. There are rarely any other species found inside the clonal ramet populations of *N. tangutorum* nebkhas.

### Methods

Plants from 12 *N. tangutorum* nebkhas of moderate size (crown within 1.5 m×1.5 m) were selected at the end of August, 2011. We excavated each nebkha to expose the layering zone and the root system (≥0.2 cm) completely. We then classified the *N. tangutorum* nebkha and its clonal ramet into four sections corresponding to the four intercardinal directions of northeast, southeast, southwest, and northwest which were respectively denoted as NE, SE, SW, and NW, the initials of the corresponding intercardinal directions. While excavating the nebkhas for each of the four aspect sections, we measured the number of the axillary buds (*a*), the number of dead ramets (*b*) (dead ramets with only a partial stem residual and that are buried by sand completely), the number of the living ramets (*c*), depth of the germination point of each axillary bud (from the surface to the depth of the germination point in cm), the number of adventitious root formation points (*N*) in every layering, and the length in cm of every layering (*L*). The ratio of total number of alive ramets and dead ramets to total axillary buds accounts for the percentage of ramet sprouting, namely the rate of ramet sprouting (*G*, in %). The number of adventitious root formation points per length of layering is called the density of adventitious root formation points (*D*, number/cm) and can measure the capacity to form adventitious roots. The above indices were used to express clonal reproduction of the layering, numerically calculated using equations (1) and (2) respectively.
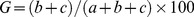
(1)

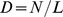
(2)


We next divided each of the layering and sprouting modules found in the different aspect sections into leaf and stem of sprouting ramet, adventitious root, and layering. To measure biomass (g), the collected materials were dried to a constant weight in the oven under 75°C. The sum of the sprouting modules' biomass was the total biomass (g). The biomass allocation of leaf modules, stem modules, and adventitious root modules (%) were measured, respectively. Layering modules were separated into two size classes of small and large; small was classified as modules with biomass ≤10 g and large was defined as modules that had a biomass >10 g.

A one-way ANOVA for the effect of aspect on depth of axillary bud germination points was conducted. A frequency distribution of germination points of axillary buds and their associated depth was also created. A MANOVA was run for the clonal reproduction and biomass allocation with the aspect and size class as fixed factors. Duncan multiple comparisons for each measure were also conducted to test the effect of the aspect and size class. Data were analyzed using SPSS16.0 software.

Because clonal reproduction and addition of biomass mainly occur in fall, spring, and summer, we selected 3 representative seasonal time periods. The 1^st^ of September, 2010 to the 15^th^ of October, 2010 represented the fall, the 1^st^ of April, 2011 to the 15^th^ of May, 2011 represented the spring, and the 16^th^ of July, 2011 to 30^th^ August, 2011 represented the summer. We measured the soil moisture and soil temperature in four aspect sections (NE, SE, NW, and SW) of *N. tangutorum* nebkhas. We sampled 4 times for each time period with an interval of 15 days. Sampling points were located at 0–10 cm soil depth in the middle slope position of the four aspect sections of one nebkha. Soil moisture was measured by comparing the weights of fresh and dried soil samples to determine percent soil moisture content. The soil temperature was measured with a soil temperature sensor (AV-10T, American Avalon, New York, USA, with a SQ2020 logger, Grant Instruments, UK). Mean values are shown in Table 1.

**Table 1:** Means of microclimate factors of *Nitraria tangutorum* nebkhas.

Aspect	Sampling Time	Soil moisture (%)	Soil Temperature(°C)
NE	Fall	1.81	18.34
	Spring	1.61	15.00
	Summer	0.91	28.10
SE	Fall	1.19	19.20
	Spring	0.89	16.40
	Summer	0.49	29.50
SW	Fall	1.22	18.92
	Spring	1.14	15.56
	Summer	0.77	28.84
NW	Fall	2.11	17.90
	Spring	1.85	14.85
	Summer	1.09	27.65

## Results

### Depth of germination point of axillary buds

**Figure 1:**
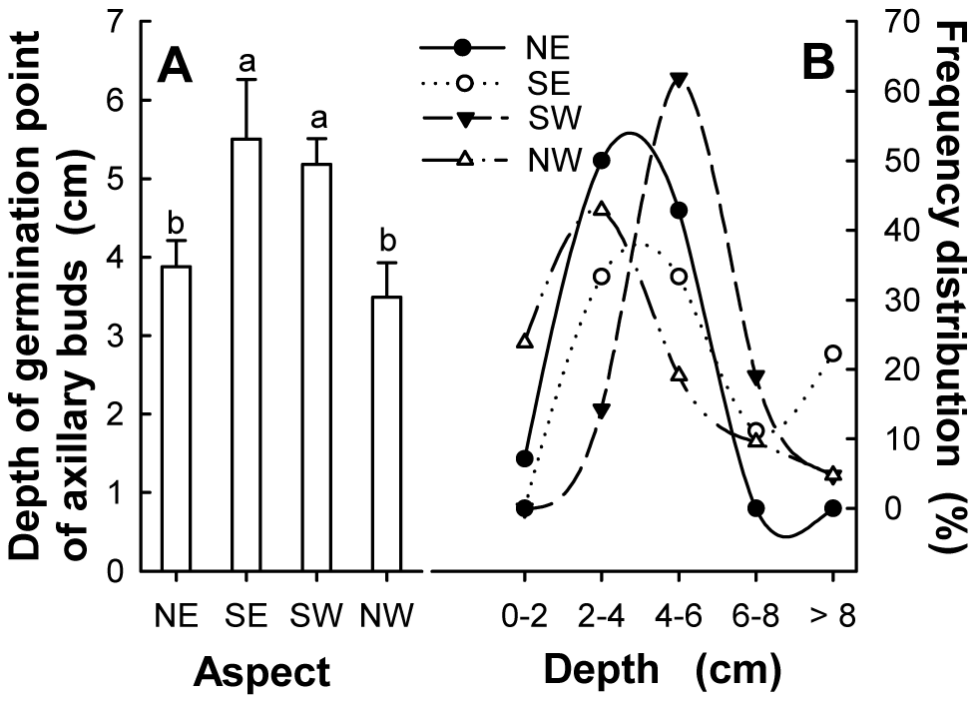
Depth of axillary bud germination points of *Nitraria tangutorum* and their frequency distribution. Treatments with different letters are significantly different (p<0.05) according to a one-way ANOVA with aspect as the factor. Error bars represent standard errors of the means.

There was a significant difference in the depth of axillary bud germination points found in layering from different aspects (ANOVA: *F* = 4.878, *P* = 0.004), which were significantly higher on the SE and SW than on the NE and NW (Fig. 1A). For depth distributions of axillary bud germination points from different aspects at the depth of 0–2 cm, the distribution in NW was greatest with little on SE and SW-facing slopes. For intervals of soil depth greater than 6 cm, SE had the greatest proportion (Fig. 1B).

### Clonal reproduction

**Figure 2:**
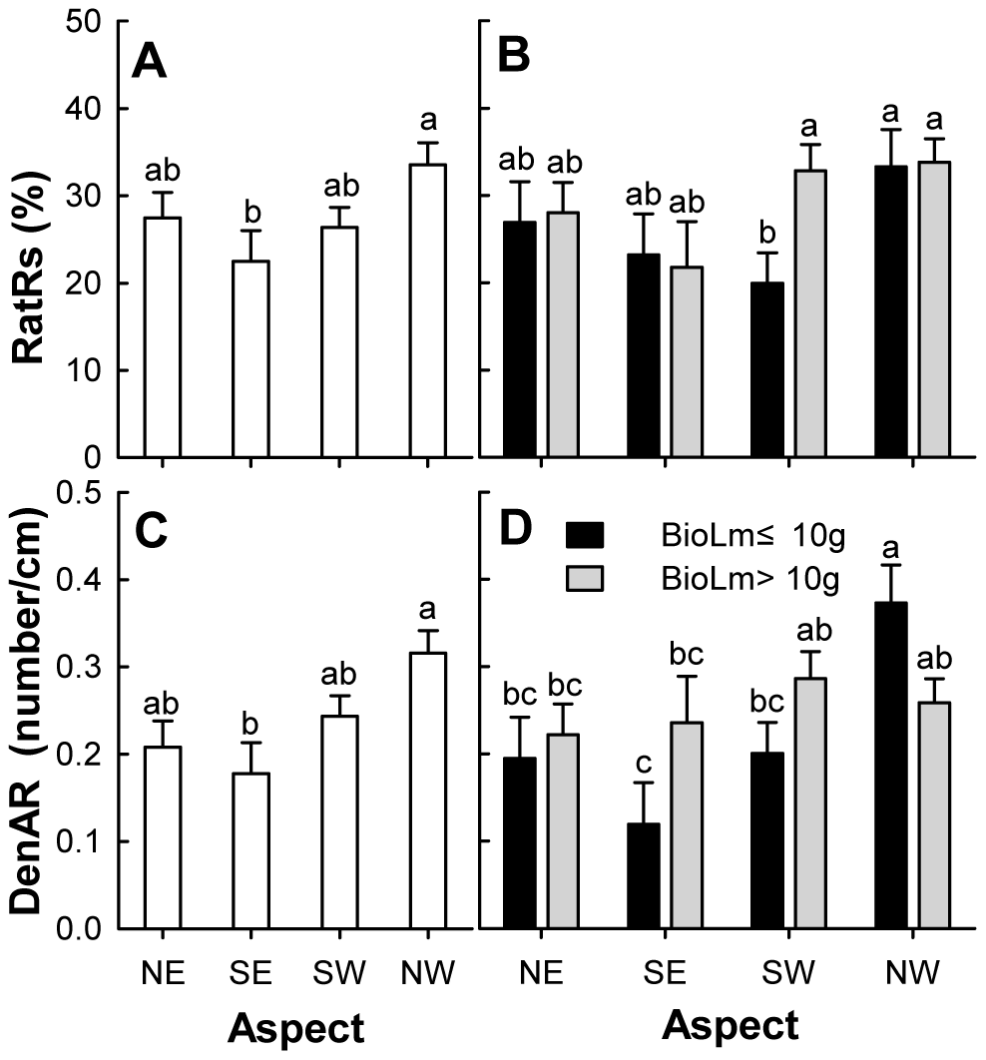
Effects of aspect and size class of layering module on clonal reproduction of *Nitraria tangutorum*. RatRs and DenAR represent the rate of ramet sprouting (%) (A and B)and density of adventitious root formation points (number/cm) (C and D), respectively. BioLm represents the layering biomass; Open bars (A and C) are grand means of four aspect sections across two size classes of layering. Grayscale bars (B and D) are means of the four aspect sections and two size classes of layering; the error bars represent standard errors of the means; the letters above the error bar are the groupings from Duncan's multiple range tests. Bars followed by different letters are significantly different at *P* = 0.05.

The clonal reproduction in different aspects was significantly different (MANOVA: Wilks' Lambda  = 0.742; *F* = 2.997; *P* = 0.009). Both the rate of ramet sprouting and the density of adventitious root formation points were significantly different with the largest values in the NW aspect (33.56% and 0.32/cm), followed by NE and then SW. The smallest values were in the SE aspect (22.49% and 0.18/cm). The size class of layering had no marked effect on clonal reproduction (MANOVA: Wilks' Lambda  = 0.963; *F* = 1.070; *P* = 0.350). Neither the rate of ramet sprouting nor density of adventitious root formation points varied for either large or small layering buried in the sand (Table 2, Fig. 2B, D).

**Table 2:** Results of ANOVA for the effect of the aspect and size class on clonal reproduction and biomass allocation of *Nitraria tangutorum*.

	Clonal reproduction				Biomass allocation					
	RatRs		DenAR		BaLeaf		BaStem		BaRoot	
Effect	*F*	*P*	*F*	*P*	*F*	*P*	*F*	*P*	*F*	*P*
Aspect (A)	2.623	0.059	4.233	0.009	2.265	0.091	2.101	0.110	5.409	0.002
Size Class (SC)	1.317	0.256	0.987	0.325	0.112	0.739	0.132	0.717	0.327	0.569
A×SC	1.665	0.185	3.556	0.020	1.169	0.330	2.894	0.043	4.843	0.005

RatRs, DenAR, BaLeaf, BaStem and BaRoot represent rate of ramet sprouting (%), density of adventitious roots formation points (number/cm), biomass allocation of leaf (%), biomass allocation of stem (%) and biomass allocation of adventitious root (%), respectively.

The interactive effect of the aspect and size class on clonal reproduction was significant (MANOVA: Wilks' Lambda  = 0.780; *F* = 2.465; *P* = 0.028). The interactive effect of these factors on the density of adventitious root formation points was significant in the ANOVA, while their effect on the rate of ramet sprouting was not (Table 2). For smaller biomass layering, the effect of aspect on clonal reproduction was significant. Both the rate of ramet sprouting and density of adventitious root formation points in the NW aspects were largest. The rate of ramet sprouting in the SW aspects was smallest, while density of adventitious root sprouting was smallest in the SE aspects. There were no significant differences in the large layering (Fig. 2B, D).

### Biomass Allocation

**Figure 3:**
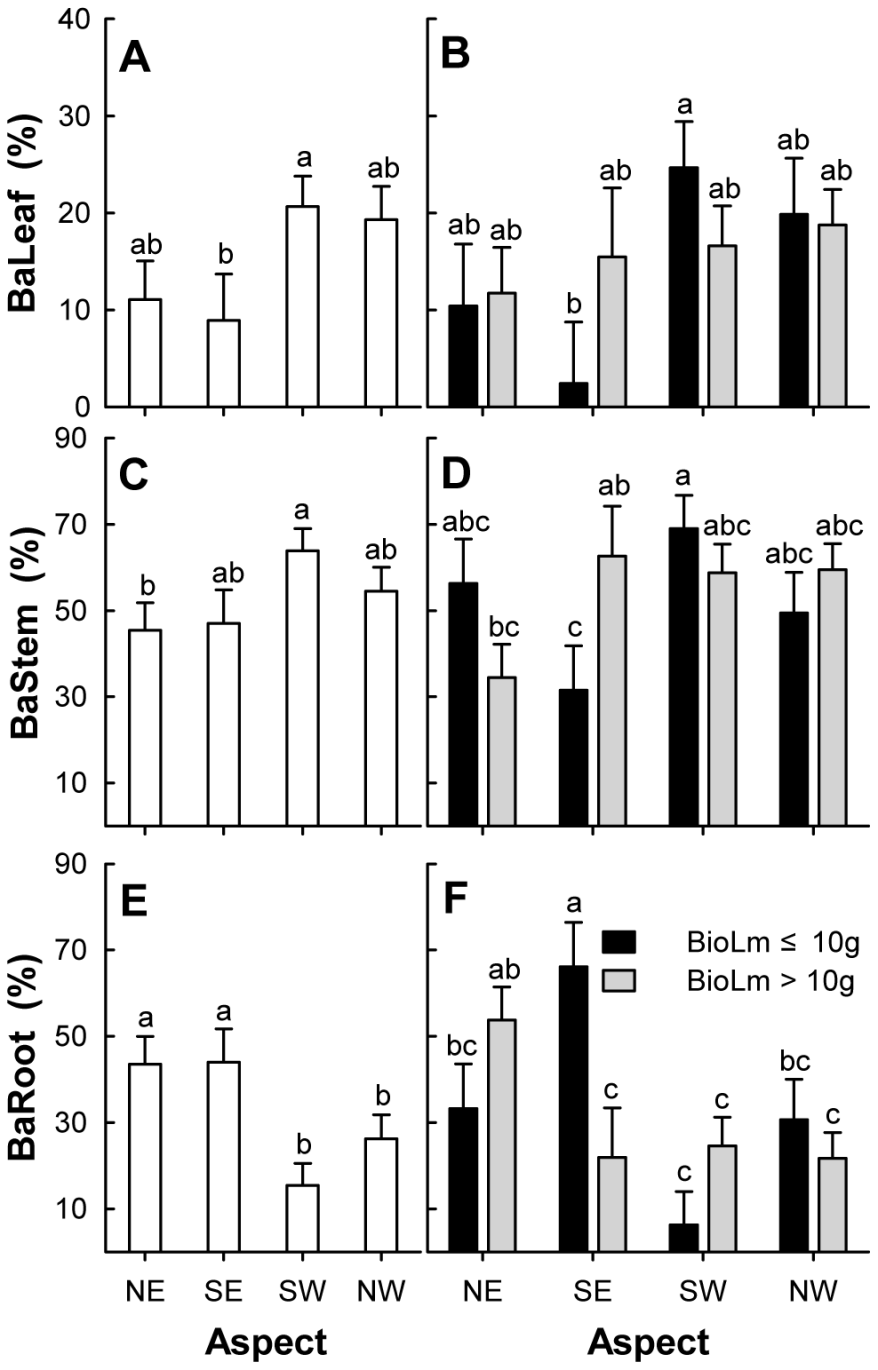
Effects of aspect and size class of layering module on biomass allocation of 3 modules of *Nitraria tangutorum*. BaLeaf, BaStem, BaRoot and BioLm represent biomass allocation of leaf (%) (A and B), biomass allocation of stem (%) (C and D), biomass allocation of root (%) (E and F) and layering biomass (G), respectively; Open bars (A, C and E) are grand means of four aspect sections across two size classes of layering. Grayscale bars (B, D and F) are means of four aspect sections and two size classes of layering combinations; Error bars represent standard errors of the means; the letters above the error bar are groupings from Duncan's multiple range tests. Bars with a different letters are significantly different at *P* = 0.05.

Aspect had a significant effect on biomass allocation of modules sprouting from the layering (MANOVA: Wilks' Lambda  = 0.752; *F* = 2.856; *P* = 0.013). The biomass allocation of leaf modules on the SW aspects was significantly higher than that on the SE aspects, with the smallest value on SE aspects being 8.94%. Biomass allocation of stem modules on the SW aspects was significantly different from that on the NE aspects (63.91% versus 45.41%, respectively). Plants on NE and SE aspect sections had significantly higher biomass allocation to adventitious roots compared to plants on SW and NW aspect sections (Table 2, Fig. 3A, C and E). Biomass allocation of the stem module in the different aspects was higher than that of the leaf module. The biomass allocation of aboveground parts of ramets was far larger than that of belowground adventitious root in the SW and NW aspect sections. The effect of layering size class on biomass allocation of the respective modules was not significant (MANOVA: Wilks' Lambda  = 0.994; *F* = 0.175; *P* = 0.840). Biomass allocation of modules sprouting from the layering of different size classes was not significantly different (Table 2).

The interaction of the aspect and size class had a significant effect on biomass allocation (MANOVA: Wilks' Lambda  = 0.777; *F* = 2.510; *P* = 0.026). In the ANOVA, the interactive effect of these factors on the biomass allocation of stem and adventitious root modules was significant (Table 2). For the small class of layering, biomass allocation to leaves and stems was the smallest on the SE aspects, whereas allocation to adventitious roots was the largest. And for the large layering, biomass allocation to adventitious roots was only significantly higher on the NE aspects when compared to the other three aspect sections.

## Discussion

The germination points of axillary buds on the layering of *N. tangutorum* were deeper on the SE and SW aspect sections of the nebkhas than on the NE and NW aspect sections. This was directly related to the high amounts of sand burial on the SE leeward sides and to the strong wind erosion on the NW windward sides. This is consistent with general law of sand movement presented in the previous studies [8], [10], [12].

In our study, the rate of ramet sprouting and the density of adventitious root formation points in layering on the northwestern windward aspects of *N. tangutorum* nebkhas were higher than layering on the southeastern leeward aspects. The biomass allocation of leaves and stems sprouting from layering on the NW aspects were higher than on the SE aspects, yet the biomass allocation of adventitious roots had the opposite pattern. These results were consistent with our first hypothesis. This suggests that the heterogeneous microenvironmental conditions resulting from different aspects play an important role in the clonal reproduction of *N. tangutorum* in nebkha dunes. The sprouting of ramets from axillary buds and the formation of adventitious roots from the root primordium would benefit from suitable microclimate conditions [43], [44], [45], [46]. We can see from Table 1 that the soil moisture is usually higher on the windward side than on the leeward side, whereas the soil temperature is lower. This may maintain soil moisture at a state suitable for the clonal growth of *N. tangutorum*. Our results are in agreement with other studies in arid land [32], [47], [48]. The fact that windblown sand movement affects the biotic communities of sand dunes has been long established [8], [10], [12]. Studies have shown that sand burial affects survival and growth of clonal plants by altering biotic and abiotic conditions [23], [25] and by creating a physical barrier that retards ramet emergence [21], [27], [29]. On the SE aspect of the *N. tangutorum* nebkha, the sprouting of buds on the layering is hindered by the thick dry sand on the surface making it difficult for new clonal ramets to form. Additionally, sand burial can promote biomass allocation to the root system of plants when there is low soil moisture [48]. The biomass allocation of a plant resistant to sand burial would favor the generation of adventitious roots as sand burial increases, even at the cost of the initial root [22]. Our results were consistent with this previous work. Change in biomass distribution patterns is an important means for plants to cope with heterogeneous habitat and as a response to changes in available resources.

In addition, the effects of aspect on clonal reproduction and on biomass allocation of leaf, stem, and adventitious root modules were greater in the small layering. This result was opposite to our second hypothesis. Sprouting of intact clones would result in the ramet increment and the adjustment of biomass allocation to ensure normal growth and development [29], [49], in which clonal integration plays an important role in the clonal growth [21], [27], [38]. The clonal growth differences were attributed to the amount of stored resources in the previous studies for the stolon or rhizome fragment [29], [33], [50]. *N. tangutorum* layering results from a cincinnal branch that is attached to the parent and buried into sand. This is different from a severed clonal fragment. One possible explanation for our result is that the traits of the bud bank on the small layering are more affected than large layering by the microenvironmental disturbances that result from the different aspects of one nebkha. Some studies have shown that the clonal growth was in relation to the size and vertical structure of buds, endogenous growth substances, and auxin application [21], [34], [36], [37], [51], [52]. This could provide a revised hypothesis for further studies of the whole-plant clonal growth. It was also inferred that the stochasticity in growth of small stems plays an important role in the establishment and persistence of clonal plants [53].

In arid and semi-arid regions, soil microenvironmental conditions such as soil water and sand movement are the main factors that limit plant growth and reproduction. Different aspects of *N. tangutorum* nebkha each have their own wind action, sand burial, and microclimate conditions that result in different soil microenvironmental conditions. Our study provides an indication that shoots buried in the windward side have a higher clonal reproduction capacity and higher biomass allocation to leaves and stems. Sand burial and lower soil moisture not only hinders the formation of new ramets but also poses a considerable threat to the survival of existing ramets on the leeward side. The information presented here should aid in the elaboration of management recommendations for conservation and preservation of *N. tangutorum* nebkhas in arid regions.
